# Supplementary material for: Potential Antiviral Options against SARS-CoV-2 Infection
Source: Viruses. 2020 Jun 13;12(6):642. doi: 10.3390/v12060642 (PMC7354438; doi:10.3390/v12060642)
Supplement: Supplementary file 1 [file viruses-12-00642-s001.pdf]

Supplementary information

Table S1. Compounds, their suppliers, catalogue numbers and AUCs.

Drug	CAS	Supplier	pur, %	Cat N	MW	Formula	AUC
Abamectin	71751-41-2	Dr. Ehrenstorfer GmbH	95.3	DRE-CA10001000	873	C95H142O28	151,97
Amiloride (EIPA)	2016-88-8	Cayman Chemical	>98	14409	300	C6H8ClN7O HCl	182,56
Amiodarone	1216715-80-8	Cayman Chemical	>99	10010668	645	C25H25D4I2NO3 HCl	173,88
Amodiaquine	86-42-0	Cayman Chemical	>95	15954	356	C20H22ClN3O	404,88
Apilimod	54550-10-0	Axon MedChem	99.7	1369	419	C23H26N6O2	585,46
Arbidol	131707-23-8	Cayman Chemical	>98.5	16933	477	C22H25BrN2O3S HCl	319,27
Artesunate	88495-63-0	Acros	n/a	460340050	384	C19H28O8	222,25
BDA-366	1909226-00-1	Selleckchem	n/a	S7849	423,5	C24H29N3O4	174,3
Bepridil	68099-86-5	Cayman Chemical	>98	19645	403	C24H34N2O HCl	285,95
Berberine	633-65-8	Cayman Chemical	>95	10006427	336	C20H18ClNO4	236,67
Brequinar	96187-53-0	Cayman Chemical	>98	24445	375	C23H15F2NO2	260,54
Bromocriptine	22260-51-1	Cayman Chemical	>98	14598	656	C32H40BrN5O5 CH3SO3H	164,57
Camostat	59721-29-8	Cayman Chemical	>98	16018	398	C20H22N4O5 CH3SO3H	88,41
Camptothecin	7689-03-4'	Cayman Chemical	>98	11694	348	C20H16N2O4	57,75
DFMO (Difluoromethylomithine)	70052-12-9	ChemCruz	>98	sc-204723	182	C6H12F2N2O2	142,66
Diphylline (7-(2,3-Dihydroxypropyl)theophylline)	479-18-5	Acros	99	115051000	524	C10H14N4O4	195,44
Emetine	7083-71-8	Cayman Chemical	>98	21048	481	C29H40N2O4 2HCl	484,19
Emodin	518-82-1	Cayman Chemical	>98	13109	270	C15H10O5	170,24
Fenretinide (4-HPR)	65646-68-6	Cayman Chemical	>98	17688	392	C26H33NO2	137,9
Formoterol	183814-30-4	Sigma-Aldrich	n/a	F0372000	420	C19H24N2O4 0.5C4H4O4 H2O	203,77
Ganciclovir	82410-32-0	Sigma-Aldrich	>99	G2536-100MG	255,23	C9H13N5O4	243,88
Glycyrrhizic acid (Glycyrrhizin)	1405-86-3	MCE	>98	HY-N0184	823	C42H62O16	235,76
Homoharringtonine	26833-87-4	Cayman Chemical	>98	14631	546	C29H39NO9	374,71
Kasugamycin	19408-46-9	Fluka	>96.5	R1114-100MG	379	C14H25N3O9	88,55
Lanatoside C	17575-22-3	MCE	99	HY-B1030	985	C49H76O20	54,25
Letermovir	917389-32-3	Cayman Chemical	>98	17556	573	C29H28F4N4O4	134,12
Lobucavir	127759-89-1	Santa Cruz	98	sc-211744	265	C11H15N5O3	153,65
Luteolin	491-70-3	Santa Cruz	>98	sc-203119	286	C15H10O6	119,07
Manidipine	89226-50-6	Cayman Chemical	>98	23614	611	C35H38N4O6	133
Maribavir	176161-24-3	MCE	>98	HY-16305	376	C15H19Cl2N3O4	145,81
Mitoxantrone	70476-82-3	Cayman Chemical	95	14842	517	C22H28N4O6 HCl	125,09
Nafamostat	82956-11-4	Cayman Chemical	>98	14837	347	C19H17N5O2 2CH3SO3H	166,53

Nelfinavir	159989-65-8	Cayman Chemical	>98	15144	664	C32H45N3O4S CH3SO3H	286,37
Novobiocin	303-81-1	Carbosynth	>94	FN59697	612	C31H36N2O11	100,73
Niclosamide	50-65-7	Dr. Ehrenstorfer GmbH	97	C15510000	327	C13H8Cl2N2O4	149,52
Obatoclax (Mesylate)	803712-79-0	MedChemExpress	99,7	HY-10969/CS-0133	413,49	$C_{21}H_{23}N_3O_4S$	385,91
Posaconazole	171228-49-2	Cayman Chemical	>95	14737	701	C37H42F2N8O4	318,22
PSI-7977 (Sofosbuvir)	1190307-88-0	MCE	100	HY-15005	530	C22H29FN3O9P	131,11
Quinine	130-95-0	Alfa Aesar	99	A10459.09	324	C20H24N2O2	140,56
Raloxifene	82640-04-8	Cayman Chemical	>98	10011620	474	C28H27NO4S HCl	199,57
Regorafenib	755037-03-7	Cayman Chemical	>98	18498	483	C21H15ClF4N4O3	24,12
Roscovitine	186692-46-6	Cayman Chemical	>98	10009569	355	C19H26N6O	137,97
Saikosaponin A	20736-09-8	Sigma Aldrich	n/a	Y0001932	780,98	C42H68O13	77
Sorafenib (BAY 43-9006)	284461-73-0	Cayman Chemical	>98	10009644	465	C21H16ClF3N4O3	33,12
Suramin	129-46-4	Acros	98	328540500	1297	C51H40N6O23S6	94,57
Tamoxifen	10540-29-1	Sigma Aldrich	>99	85256-50MG	372	C26H29NO	54,67
Zanamivir	139110-80-8	Cayman Chemical	≥98	15123	332,3	C12H20N4O7	113,54
Genistin	529-59-9	Cayman Chemical	≥98	14174	432,4	C21H20O10	54,1
Pleconaril	153168-05-9	Cayman Chemical	≥98	28461	381	C18H18F3N3O3	141,75
Salinomycin	53003-10-4	MedChem Express	≥98	HY-15597	751	C42H70O11	334,04
Monensin	22373-78-0	Cayman Chemical	≥98	16488	671	C36H61O11 • Na	254,31
Enoxacin	74011-58-8	Alfa Aesar	n/a	J61912	320	C15H17FN4O3	118,23
Hexachlorophene	70-30-4	Cayman Chemical	≥98	CAYM23948	407	C13H6Cl6O2	122,15
SNS-032	345627-80-7	Selleckchem	≥98	S1145	380,5	C17H24N4O2S2	96,25
Trametinib	871700-17-3	MedChemExpress	9944	HY-10999/CS-0060	615,39	$C_{26}H_{23}FIN_5O_4$	117,53
Ciclesonide	126544-47-6	Sigma-Aldrich	98	SML1955-5MG	540,69	C32H44O7	157,15
Pimodivir	1629869-44-8	MedChemExpress	99	HY-12353A/CS	399,39	$C_{20}H_{19}F_2N_5O_2$	126,91
Ouabain octahydrate	11018-89-6	Sigma-Aldrich	95	O3125-250MG	728,77	C29H44O12・8H2O	41,93
Tilorone (dihydrochloride)	27591-69-1	MedChemExpress	99	HY-B1080/CS-4636	483,47	$C_{25}H_{36}Cl_2N_2O_3$	83,72
Staurosporine	62996-74-1	Sigma-Aldrich	98	S4400-1MG	466,53	C22H18O12	15,75
Chicoric acid	6537-80-0	Sigma-Aldrich	95	C7243-10MG	474,37	C22H18O12	93,1
Ascorbic acid	50-81-7	Sigma-Aldrich	98	A4544-25g	176,12	C6H8O6	108,5
Acetylsalicylic acid	50-78-2	Acros Organics	99	158180500	180	C9H8O4	131,04
Aciclovir (acycloguanosine)	59277-89-3	Acros Organics	98	445240100	225	C8H11N5O3	124,95
Azacytidine	320-67-2	Acros Organics	99	226620500	244	C8H12N4O5	228,06
Azithromycin	83905-01-5	Santa Cruz Biotechnology	>95	SC-254949	749	C38H72N2O12	119,42
BCX4430 (galidesivir)	249503-25-1	MedChem Express	>99	HY-18649	265	C11H15N5O3	94,08

Bortezomib (PS-341)	179324-69-7	Selleckchem	99	S1013	384	C19H25BN4O4	68,67
Brincidofovir (CMX001)	444805-28-1	MedChem Express	>98	HY-14532	562	C27H52N3O7P	126,49
Caffeine	58-08-2	Acros Organics	>98	108160100	194	C8H10N4O2	147,28
Chloroquine phosphate	50-63-5	Sigma-Aldrich	>99	PHR1258	516	C18H26ClN3 2H3PO4	165,9
Cidofovir	113852-37-2	Cayman Chemical	95	CAYM13113	279	C8H14N3O6P	144,69
Clofarabine	123318-82-1	Sigma-Aldrich	98	C7495-5MG	303,68	C10H11ClFN5O3	112,42
Cyclosporine A	59865-13-3	Acros Organics	98	457970010	1202	C62H111N11O12	146,37
Dasatinib	302962-49-8	Sigma-Aldrich	>99	CDS023389	488	C22H26ClN7O2S	135,45
Dibucaine	61-12-1	Sigma-Aldrich	99	D0638	380	C20H29N3O2 HCl	214,34
Doxycycline hyclate	24390-14-5	Sigma-Aldrich	>98	D9891	513	C22H24N2O8 HCl 0.5H2O 0.5C2H6O	143,99
Erlotinib	183319-69-9	Sigma-Aldrich	>99	CDS022564	430	C22H24ClN3O4	103,46
Ezetimibe	163222-33-1	Cayman Chemical	>98	CAYM16331	409	C24H21F2NO3	138,39
Esomeprazole	668985-31-7	Sigma-Aldrich	>98	E7906	713	C34H36MgN6O6S2 H2O	177,52
Famciclovir	104227-87-4	Sigma-Aldrich	>98	F7932	321	C14H19N5O4	149,52
Favipiravir (T-705)	36791-04-5	Selleckchem	98	S7975	244	C8H12N4O5	136,64
Fluoxetine	56296-78-7	Santa Cruz Biotechnology	>98	SC-201125	345	C17H18F3NO HCl	104,37
Fluvastatin	93957-55-2	Acros Organics	>97	458010010	433	C24H25FNNaO4	154,14
Foscarnet	63585-09-1	Selleckchem	>98	S3076	192	CNa3O5P	127,26
Gefitinib	184475-35-2	Santa Cruz Biotechnology	>99	sc-202166	447	C22H24ClFN4O3	188,86
Gemcitabine	122111-03-9	Sigma-Aldrich	98	G6423	300	C9H11F2N3O4 ·HCl	92,89
Hydroxychloroquine	747-36-4	Santa Cruz Biotechnology	>97	sc-215157	434	C18H26ClN3O•H2SO4	147,63
Imatinib	220127-57-1	Sigma-Aldrich	>98	SML1027	590	C29H31N7O · CH4O3S	177,8
Indomethacin	53-86-1	Acros Organics	>97	458030050	358	C19H16ClNO4	104,51
Itraconazole	84625-61-6	Acros Organics	99	452870050	706	C35H38Cl2N8O4	110,18
Ivermectin	70288-86-7	Alfa Aesar	>90	J62777	875	C48H74O14	66,08
Lamivudine	134678-17-4	Selleckchem	>99	S1706	229	C8H11N3O3S	106,75
Leflunomide	75706-12-6	Santa Cruz Biotechnology	>99	SC-202209	270	C12H9F3N2O2	47,95
Lopinavir	192725-17-0	Sigma-Aldrich	>98	SML1222	629	C37H48N4O5	157,36
Lovastatin	75330-75-5	Sigma-Aldrich	>99	PHR1285	405	C24H36O5	204,26
Memantine	41100-52-1	Acros Organics	99	298080010	216	C12H21N HCl	115,78
Metformin (1,1-Dimethylbiguanide)	214-230-6	Alfa Aesar	>97	J63361	166	C4H11N5 HCl	116,76
Minocycline	13614-98-7	Cayman Chemical	>98	CAYM14454	458	C23H27N3O7 HCl	119,14
Mycophenolic acid	24280-93-1	Acros Organics	98	459380010	320	C17 H20 O6	103,74
Nitazoxanide	55981-09-4	Cayman Chemical	>95	CAYM13692	307	C12H9N3O5S	120,33
Omeprazole	73590-58-6	Sigma-Aldrich	>99	O104	345	C17H19N3O3S	139,02
Oritavancin	192564-14-0	Sigma-Aldrich	>97	SML1586	1989	C86H97Cl3N10O26 2H3PO4	163,24
Pentosan polysulfate	9062-57-1	MedChem Express	>98	HY-A0203	602	C10H18O21S4	123,06
Pirlindole mesylate	60762-57-4	Santa Cruz Biotechnology	>99	SC-203664	322	C15H18N2 CH3SO3H	120,4

Rapamycin	53123-88-9	Fisher scientific	>98	BP2963	914	C51H79NO13	46,9
Ribavirin	36791-04-5	Acros Organics	98	460480010	244	C8H12N4O5	134,26
Ritonavir	155213-67-5	Cayman chemical co	>98	CAYM13872	721	C37H48N6O5S2	137,69
Simvastatin	79902-63-9	Acros Organics	98	458840010	419	C25H38O5	163,03
Teicoplanin	61036-62-2	Sigma-Aldrich	>99	Y0001102	1879	Variable	145,39
Telavancin	372151-71-8	Adooq Biosc	>98	A12698	1755	C80H106Cl2N11O27P	139,65
Tenofovir disoproxil fumarate	202138-50-9	Acros Organics	98	461250010	636	C19H30N5O10P C4H4O4	137,2
Topotecan	119413-54-6	Santa Cruz Biotechnology	99	SC-204919	458	C23H23N3O5 HCl	124,6
Trifluridine	70-00-8	Selleckchem	>99	S1778	296	C10H11F3N2O5	107,17
Valaciclovir	124832-27-5	Sigma-Aldrich	>99	Y0001225	361	C13H20N6O4 HCl	134,61
Verapamil	152-11-4	Acros Organics	99	329330010	491	C27H38N2O4 HCl	134,26
Bromhexine HCl	611-75-6	Sigma-Aldrich	98	17343-25G	412,59	C14H20Br2N2 ·HCl	124,95
Bromcriptine mesilate	22260-51-1	Sigma-Aldrich	n/a	Y0000677	750,7	C32H40BrN5O5 · CH4SO3	59,71
Cefoperazone acid	62893-19-0	Santa Cruz Bio	>98	SC-204677	545,7	C25H27N9O8S2	22,05
Moxalactam sodium salt	64963-12-4	Sigma-Aldrich	n/a	M8158-1G	564,4	C25H27N9O8S2	18,13
Anisomycin	22862-76-6	Sigma-Aldrich	>98	A9789-5mg	265,3	C14H19NO4	169,33
Benzatropine	132-17-2	Sigma-Aldrich	>98	SML0847-500mg	403,53	C21H25NO · CH3SO3H	208,11
Diltiazem	33286-22-5	Sigma-Aldrich	n/a	D1980000	414,519	C22H26N2O4S	99,61
Teriflunomide	15345-81-8	Sigma-Aldrich	>98	SML0936-10MG	270,2	C12H9F3N2O2	72,87
Clomiprade hydrochloride	17321-77-6	Sigma-Aldrich	>98	C7291-1G	351,31	C19H23ClN2 ·HCl	74,69
Chlorpromazine hydrochloride	69-09-0	Sigma-Aldrich	>98	C8138-5G	356,33	C17H19ClN2S ·HCl	86,52
Quinacrine dihydrochloride	69-05-6	Sigma-Aldrich	>90	Q3251-25G	472,88	C23H30ClN3O ·2HCl	105,63
Toremifene	89778-27-8	Sigma-Aldrich	>98	T7204-5MG	598,1	C32H36ClNO8	27,02
Ranitidine	66357-35-5	Sigma-Aldrich	n.a.	R101-1G	350,9	C13H22N4O3S ·HCl	94,08
Stanozolol	10418-03-8	Sigma-Aldrich	n.a.	S1250000	328,5	C21H32N2O	161,7
Bumetanide	28395-03-1	Sigma-Aldrich	>98	B3023-250MG	364,4	C17H20N2O5S	87,85
Dalbavancin	171500-79-1	Sigma-Aldrich	>98	SML2378-5MG	1816	C88H101Cl3N10O28	136,29
Remdesivir	1809249-37-3	Cayman	>98	30354	602,6	C27H35N6O8P	290,29
Trametinib	871700-17-3	Cayman	>98	16292	615,4	C26H23FIN5O4	100,59
Acetylcysteine	616-91-1	Cayman	>98	20261	163,2	C5H9NO3S	43,47
Verdinexor	1392136-43-4	Cayman	>98	26171	442,3	C18H12F6N6O	83,93
Mefloquine	51773-92-3	Sigma-Aldrich	n.a.	M0253000	414,8	C17H17ClF6N2O	189.28

Table S2. Results of neutralization and ELISA assays.

**Sample N**	**s/co, IgM**	**s/co, IgG**	**SSS**	**Virus**
10	14,35	2,11	17,8	SARS-CoV-2
11	2,25	2,92	37,6	SARS-CoV-2
12	3,59	2,95	5	SARS-CoV-2

13	1,44	3,02	16,1	SARS-CoV-2
14	0,48	0,26	6,1	SARS-CoV-2
15	0,53	3,77	2,5	SARS-CoV-2
16	0,43	0,39	0,9	SARS-CoV-2
17	0,43	2,56	17,1	SARS-CoV-2
18	0,53	1,10	5,7	SARS-CoV-2
19	0,48	0,55	0,3	SARS-CoV-2
26	0,43	0,94	0	SARS-CoV-2
27	0,48	1,33	1,8	SARS-CoV-2
28	0,91	3,02	7,4	SARS-CoV-2
29	0,41	1,33	0	SARS-CoV-2
30	0,41	2,05	0,8	SARS-CoV-2
31	0,48	1,85	2,6	SARS-CoV-2
32	0,62	2,31	2,9	SARS-CoV-2
81	0,53	0,52	0	HCoV-HKU1
82	0,48	0,58	0,5	HCoV-NL63
83	0,53	0,34	0	HCoV-NL63
86	0,48	0,48	0,5	HCoV-OC43
88	0,48	0,75	0,4	HCoV-OC43
91	0,48	0,61	2,6	HCoV-229E
92	1,20	0,51	1,4	HCoV-229E
72	0,35	0,27	0	Healthy blood donor
73	0,35	0,45	0	Healthy blood donor
74	0,39	0,45	0	Healthy blood donor
75	0,39	0,55	0	Healthy blood donor
76	0,39	0,30	0	Healthy blood donor
77	0,43	0,18	0	Healthy blood donor
78	0,39	0,36	0	Healthy blood donor
79	0,35	0,85	0	Healthy blood donor

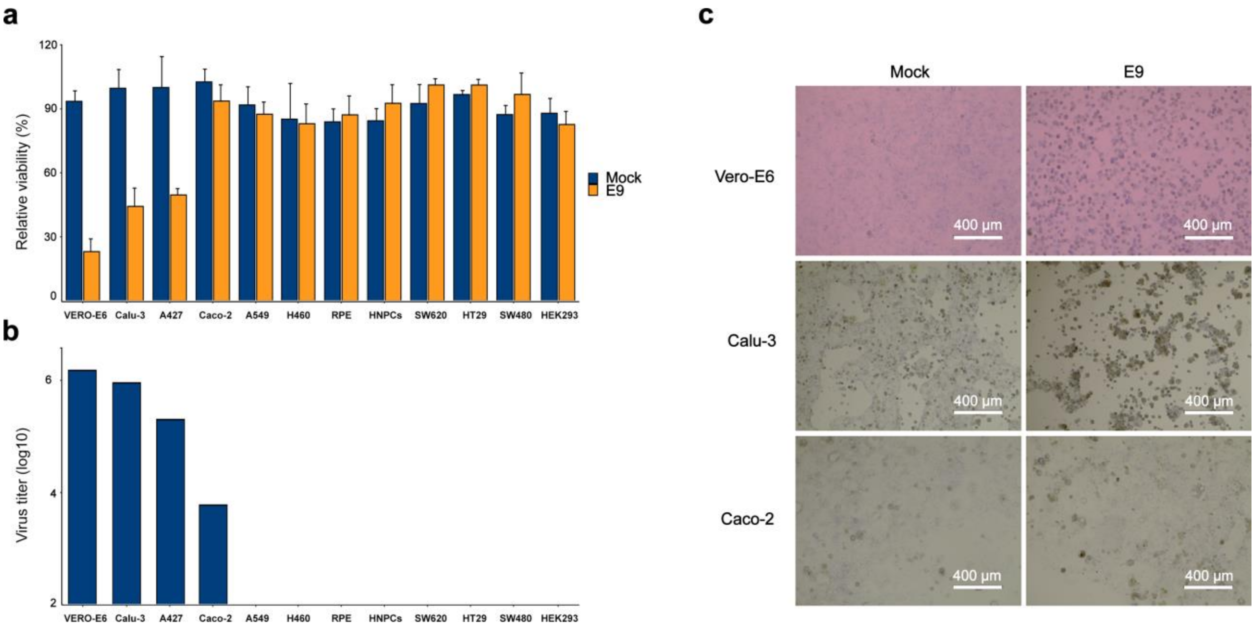

Figure S1. Propagation of HCoV-19/Norway/Trondheim-E9/2020 in cell cultures. (a) Cell lines were infected with the virus and cell viability were measured. (b) Viruses amplified in the cells were quantified by plaque assay. (c) Viability of mock- and virus-infected cells were visualized by microscopy (bright field).

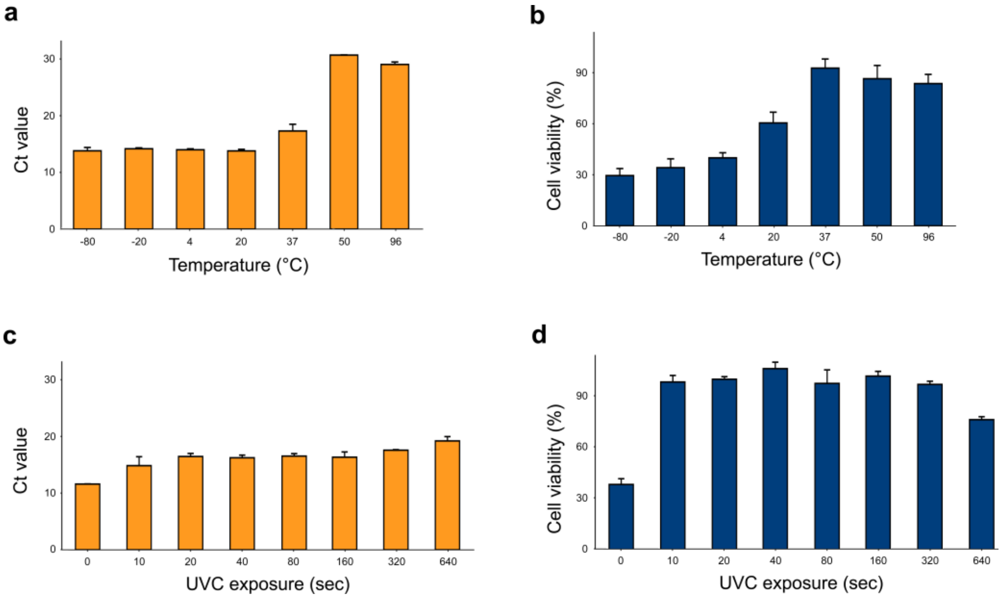

Figure S2. Effect of temperature and UV radiation on infectivity of HCoV-19/Norway/Trondheim-E9/2020 strain. (a) The virus was incubated at -80, -20, 4, 20, 37, and 50 °C for 48 h or at 96 °C for 10 min. The thermostability of viral RNA was analysed by RT-qPCR. (b) Vero-E6 cells were subsequently infected with the virus. After 72 h cell viability was measured. Mean ± SD, n = 3. (c) The virus was exposed to UVC for different times. The stability of viral RNA was analysed by RT-qPCR. Mean ± SD, n = 3. (d) Vero-E6 cells were subsequently infected with the virus. After 72 h cell viability was measured. Mean ± SD, n = 3.

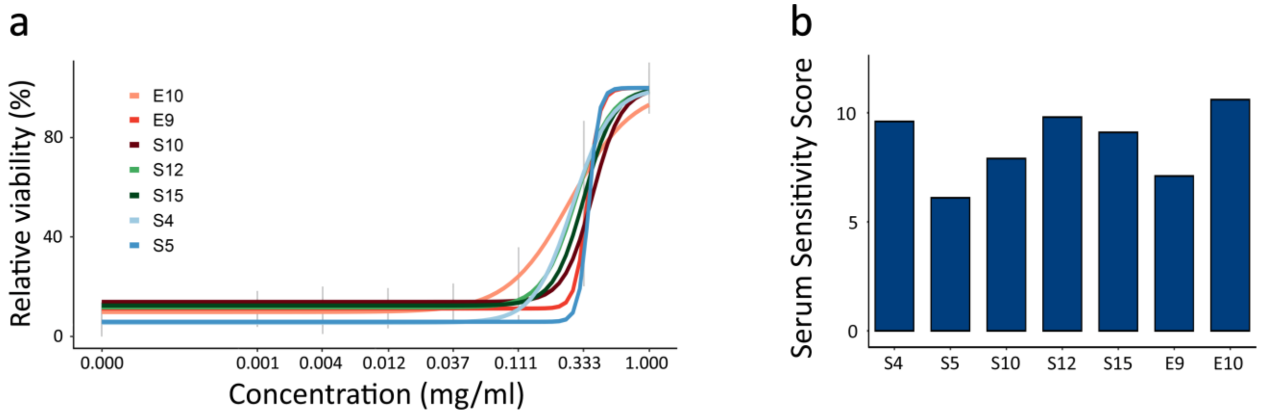

Figure S3. The effect of serum from patient recovered from COVID-19 on viability of Vero-E6 cells infected with 7 SARS-CoV-2 strains.

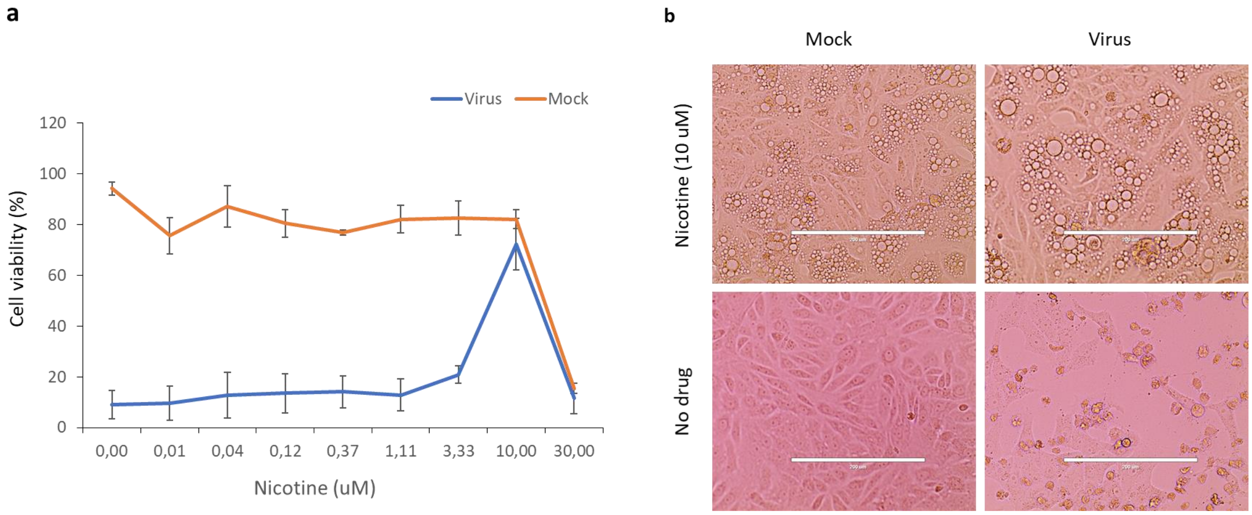

Figure S4. The effect of nicotine on viability and morphology of mock- and SARS-CoV-2-infected Vero-E6 cells. (**a**) Cells were treated with increasing concentrations of a compound and infected with HCoV-19/Norway/Trondheim-E9/2020 strain (moi, 0.1) or mock. After 72 h the viability of the cells was determined using the CTG assay. Mean ± SD; n = 3. (**b**) Cells were treated as for (a). 72 h after infection cells were imaged using a bright-field microscopy. Scale bar, 200 µm.

a

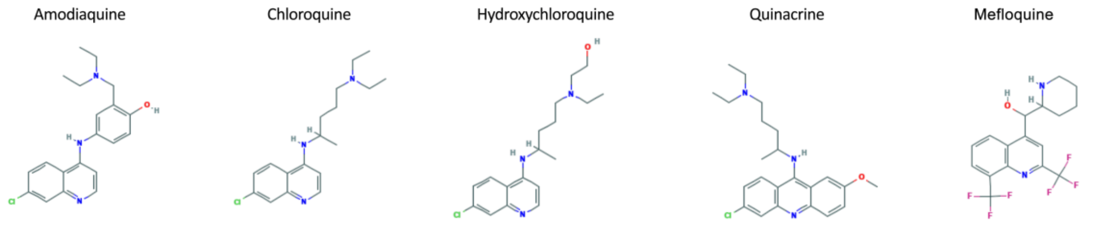

b

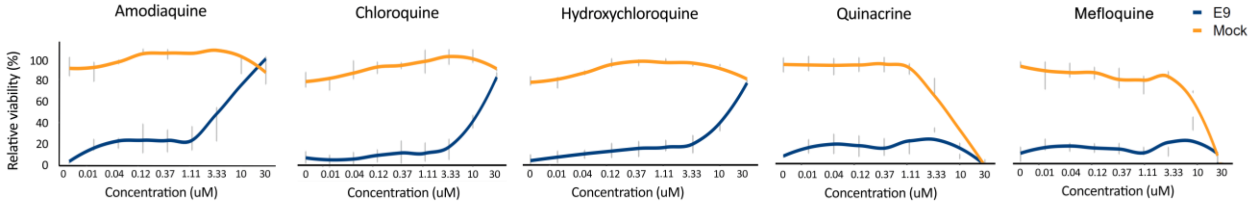

c

Drug	CC50, uM	EC50_1, uM	EC50_2, uM	SI_1	SI_2
Amodiaquine	>30	4,2±0,2	5,0±0,7	>7,1	>6,0
Chloroquine	>30	15,5±1,0	18,0±1,6	>1,9	>1,7
Hydroxychloroquine	>30	13,7±0,8	16,2±1,2	>2,2	>1,9
Quinacrine	13,9±0,3	n.a.	8,4±2,3	n.a.	1,7
Mefloquine	16.1±0,1	n.a.	n.a.	n.a.	n.a.

Figure S5. Comparison of anti-SARS-CoV-2 activities of amodiaquine and its analogues. (**a**) Structure of amodiaquine and its analogues. (**b**) Anti-SARS-CoV-2 activity of amodiaquine and its analogues in Vero-E6 cells. Cells were treated with increasing concentrations of a compound and infected with HCoV-19/Norway/Trondheim-E9/2020 strain (moi, 0.1) or mock. After 72 h the viability of the cells was determined using the CTG assay. Mean ± SD; n = 3. (**c**) Table showing half-maximal cytotoxic concentration (CC_{50}), the half-maximal effective concentration (EC_{50}), and selectivity indexes ($SI=CC_{50}/EC_{50}$) for amodiaquine and its analogues calculated from CTG and plaque assays. Mean ± SD; n = 3.

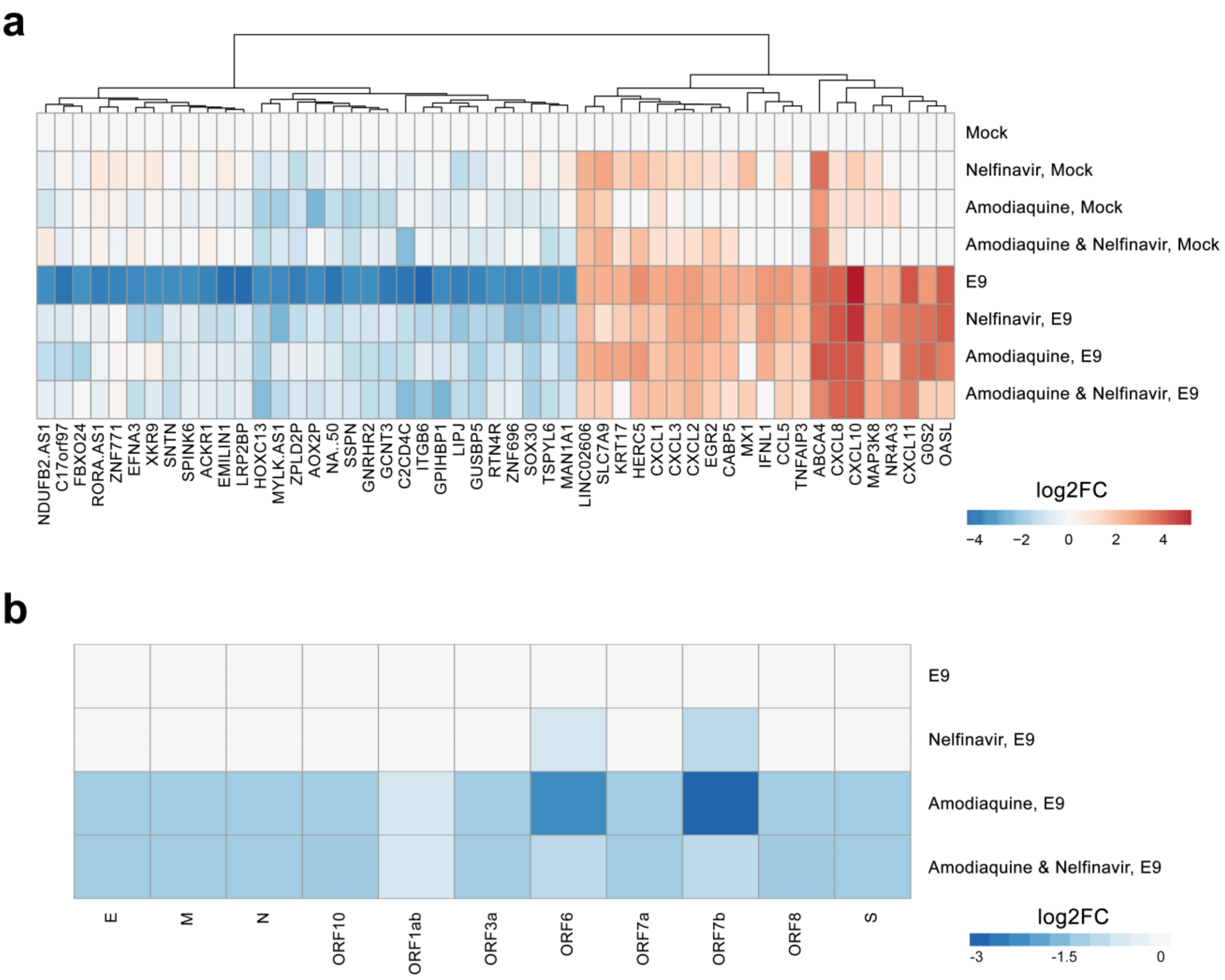

Figure S6. Transcriptomic analysis of mock- and SARS-CoV-2-infected Vero-E6 cells treated with nelfinavir, amodiaquine or both drugs. (**a**) Heatmap depicting the most significantly up- and down-regulated human genes in mock- and SARS-CoV-2 infected (moi 0,1) Vero-E6 cells treated with 3 µM nelfinavir, 5 µM amodiaquine or both drugs for 24 h. Colored bar represents the log2-transformed fold change (FC) values normalized to mock control. (**b**) Heatmap showing log2-transformed expression of SARS-CoV-2 mRNAs in Vero-E6 cells treated with 3 µM nelfinavir, 5 µM amodiaquine or both drugs. Coloured bar represents the log2-transformed fold change (FC) values normalized to SARS-CoV-2 infected cells without drug treatment.
